# Mechanical acupuncture at HT7 attenuates alcohol self-administration in rats by modulating neuroinflammation and altering mPFC-habenula-VTA circuit activity

**DOI:** 10.3389/fnbeh.2024.1455622

**Published:** 2024-10-30

**Authors:** Su Yeon Seo, Se Kyun Bang, Suk Yun Kang, Seong Jin Cho, Kwang-Ho Choi, Yeonhee Ryu

**Affiliations:** ^1^Department of Oriental Medicine Research, Korea Institute of Oriental Medicine, Daejeon, Republic of Korea; ^2^Department of Korean Convergence Medical Science, University of Science and Technology (UST), Daejeon, Republic of Korea

**Keywords:** alcohol, acupuncture, habenula, microglia, BDNF

## Abstract

**Introduction:**

Alcohol use disorder is a chronic disorder with significant limitations in pharmacological treatments, necessitating the exploration of non-pharmacological interventions.

**Methods:**

We used a model of alcohol self-administration (10% v/v) to analyze behavioral, neurochemical, and signaling mechanisms.

**Results:**

Our findings demonstrate that stimulation of the HT7 acupuncture point significantly decreased the frequency of active lever presses in rats self-administering alcohol (*p* < 0.05). Alcohol self-administration increased microglial activity and sigma 1 receptor expression in the habenula (Hb), while HT7 stimulation mitigated these effects, decreasing microglial activity and sigma 1 receptor levels (*p* < 0.05). Additionally, alcohol self-administration reduced brain-derived neurotrophic factor (BDNF) expression in the medial prefrontal cortex (mPFC) and increased tyrosine hydroxylase (TH) levels in the ventral tegmental area (VTA) (*p* < 0.05). HT7 stimulation reversed these alterations by increasing BDNF expression in the mPFC and decreasing TH levels in the VTA (*p* < 0.05). Further investigation revealed that BDNF microinjection into the mPFC inhibited sigma 1 receptor activity in the Hb, while microglial inhibition in the Hb decreased TH expression in the VTA (*p* < 0.05). The administration of the microglial inhibitor MINO to the Hb also reduced alcohol self-administration (*p* < 0.05).

**Discussion:**

These results suggest that HT7 stimulation regulates the mPFC-Hb-VTA circuit, leading to decreased alcohol-seeking behavior. Our study demonstrates that HT7 acupuncture can modulate the mPFC-Hb-VTA circuit, providing a potential non-pharmacological treatment for alcohol-seeking behavior by influencing microglial activity, sigma 1 receptor expression, and TH levels. These findings contribute to a deeper understanding of the neural mechanisms underlying acupuncture’s therapeutic effects on alcohol use disorder.

## 1 Introduction

Alcohol consumption has been linked to various social and economic problems throughout society. Unlike other illicit substances of abuse, alcohol is both legal and widely accepted, making its use exceptionally prevalent within our society. Excessive alcohol consumption causes variety of neuropsychiatric disorders, including depression, anxiety, and insomnia. According to the World Health Organization (2019), 4.2 billion people were current drinkers in 2019. Alcohol use disorder is also a trend that increases yearly. To date, there are only 4 pharmacological treatments approved in Europe for alcohol use disorders (AUDs): acamprosate, disulfiram, naltrexone, and nalmefene (Røsner and Soyka, 2019). Although these pharmacological treatments reduce cravings for heavy drinking and improve abstinence rates, the problem of side effects cannot be avoided. Because pharmacological treatment has clear limitations, various non-pharmacological treatments have been proposed to alleviate alcohol use disorder. For example, include neurostimulation, meditation, cognitive behavioral therapy, and acupuncture.

Acupuncture, a well-known form of alternative therapy, has been used for treating various disorders, including pain, specific drug addiction, and withdrawal syndromes. Among them, the name of acupuncture point HT7 (Shenmen) means the “gate of spirit,” and this acupuncture point is linked to traditional treatment for disorders such as amnesia, stupor, mania, insomnia, irritability, and addiction (Khongrum and Wattanathorn, 2017; Lai et al., 2016). For this reason, many clinical and preclinical studies have shown the effect mechanism of HT7 acupuncture point stimulation. For example, HT7 stimulation reduces alcohol self-administration by suppressing GABA neuron activity in the ventral tegmental area (VTA; Yang et al., 2010; Lee et al., 2014; Park et al., 2012). In addition, HT7 acupuncture point stimulation improves alcohol-induced anxiety and alcohol intake via the modulation of corticotropin-releasing factor and neuropeptide Y in the amygdala (Zhao et al., 2014). Recently, HT7 stimulation was shown to attenuate alcohol dependence through the activation of endorphinergic input to the nucleus accumbens (NAc) from the arcuate nucleus (Chang et al., 2019). There is also much evidence that HT7 acupoint stimulation modulates not only neuronal but also glial activity. The HT7 stimulation was shown to inhibit microglial activation and modulate astrocyte proliferation in a pain model (Liang et al., 2016). In this way, the addiction-relieving effect of acupuncture treatment appears through various brain regions and the activity of various cell types, and various studies are currently underway on the addiction-relieving impact of acupuncture treatment.

Much research has focused on the mesolimbic dopamine system in relation to various drug addictions, including alcohol use disorder. However, recent studies on drug addiction have expanded to encompass additional brain regions beyond the mesolimbic dopamine system. Efforts have been made to modulate dopamine expression in the VTA by targeting various brain regions. One area that has recently been receiving attention is the habenula (Hb). The lateral habenula (LHb) sends inhibitory projections to the VTA, thereby influencing the activity of dopaminergic neurons. The LHb receives relay information from the limbic forebrain and basal ganglia structures to basically all midbrain monoaminergic centers, including the serotonergic raphe and dopaminergic midbrain nuclei (Bernard and Veh, 2012; Poller et al., 2011), controlling mood and emotions and playing a critical part in the brain’s response to reward. The inhibition of VTA dopaminergic neurons by the LHb is believed to contribute to the encoding of aversive stimuli and the suppression of reward-related behavior. Recently, evidence has suggested that acupuncture stimulation regulates the activity of neurons in the Hb region. Electroacupuncture was shown to alleviate hyperalgesia during ethanol (EtOH) withdrawal through a mechanism involving mu-opioid receptors in the Hb (Li et al., 2017). The effect of HT7 stimulation involved in inhibiting cocaine-induced hyperlocomotion was shown to be induced by the c-fos activity of the dorsal columnal course and LHb (Chang et al., 2017). Many studies have shown that acupuncture is effective in the treatment of mood and emotional disorders and drug abuse. However, despite the presence of many microglia in Hb, it is not known how acupuncture stimulation regulates microglial activity in the Hb region.

Meanwhile, neurotransmitters, neurohormones, and growth factors also affect dopamine expression in the VTA. Typically, growth factors regulate responses to drugs of abuse, including alcohol. Brain-derived neurotrophic factor (BDNF) is a representative growth factor. BDNF is expressed in various brain regions, including the hippocampus, NAc, amygdala, VTA, and prefrontal cortex. The expression of BDNF related to alcohol use disorder has been actively studied in the mPFC. Aberrant BDNF signaling in the medial prefrontal cortex (mPFC) contributes to the long-lasting structural changes seen in alcohol use disorder (Bramham, 2008; Poo, 2001); the inhibition of BDNF expression in the mPFC increased alcohol self-administration, and an increase in BDNF expression decreased alcohol self-administration. There are many strategies to increase BDNF in the brain, and acupoint stimulation is one method. According to a recent study, acupuncture stimulation has been reported to increase BDNF in various brain regions, including the mPFC (Liang et al., 2012; Zhang et al., 2020). Our previous study also confirmed an increase in BDNF in various brain regions due to HT7 acupuncture stimulation. In a caffeine-induced sleep disorder model, endoplasmic reticulum stress was lowered through an increase in BDNF in the medial septum region (Seo and Ryu, 2022). In a menopausal depression model, NPY expression was regulated by increasing BDNF in the hippocampus through SP6 stimulation (Seo et al., 2018). There is a lack of research on whether HT7 stimulation can induce increases in BDNF within the brain in alcohol self-administration models.

This study investigated whether acupuncture had a significant effect on reducing alcohol consumption in a rat model of alcohol self-administration. We confirmed the activity of microglia in the Hb and clarified the mechanisms underlying the upregulation and downregulation of Hb activity by acupuncture stimulation.

## 2 Materials and methods

### 2.1 Animals

Adult male Wistar rats (250–300 g) were acquired from Orient Bio (Seongnam, Korea). The rats were housed in colony cages with free access to food and water and maintained on a 12-h light/12-h dark cycle throughout the study period. All the procedures used in the present study were reviewed and approved by the Animal Care and Use Committee at the Korea Institute of Oriental Medicine (KIOM, Daejeon, Korea), reference number #20–017, and conformed to National Institutes of Health (NIH) guidelines (NIH publication number 86–23, revised 1985). Every effort was made to minimize animal distress and discomfort and to reduce the number of animals used in the study.

### 2.2 Alcohol self-administration

Alcohol self-administration was performed in an operant chamber (measuring 31.8 cm × 25.4 cm × 26.7 cm, MED Associates Inc., Georgia, VT, USA) equipped with two response levers and a house light. When the experimental animals pressed the active lever once, a 0.1-ml drop of alcohol solution was dispensed onto a dish kept on the center panel of the operant chamber, and the light was turned on during the self-administration session. On the other hand, when the animals pressed the active lever, the response was recorded, but there was no significant consequence. The animals underwent behavioral training 5 days a week for 30 min a day. Using the modified sucrose-fading method described previously, the animals were trained to self-administer alcohol orally (Kang et al., 2017). Briefly, the rats gradually became accustomed to the behavior during the training period (to advance the process of approaching the active lever and the dish). To facilitate the essential process of pressing the active lever, 20% sucrose solution was initially used for self-administration training. The rats were trained in a daily 30 min session to press a lever for a 20% (w/v) sucrose solution on a fixed ratio (FR1) reinforcement schedule until an acquisition criterion of 100 lever presses for three consecutive sessions was achieved. After the animals learned to push the active lever firmly to keep drinking the 20% sucrose solution, 10% sucrose solution was used for the next active lever response step. When a stable baseline reaction was established, the concentration of sucrose in the solution was gradually reduced to 0%, and the alcohol concentration was ultimately increased to 10%. The detailed steps were as follows: 1 week with 2% alcohol in 10% sucrose, 1 week with 5% alcohol in 10% sucrose, and 1 week with 10% alcohol in 5% sucrose. Finally, 10% alcohol without sucrose was presented as the addiction reinforcer. After rats showed a stable response to the 10% alcohol solution and met an established criterion for alcohol baseline response, treatment with acupuncture stimulation was initiated. The criteria for the alcohol baseline were determined by the mean value of three consecutive alcohol intakes using the animals that showed a stable response to 10% alcohol, with a variation of less than 20%. The total number of alcohol intake sessions was determined for each treatment group over 3 days, during which the induced alcohol intake was stable, and the amount of alcohol intake was averaged. The alcohol intake of the ETOH group was 10.52 g/kg per 40 levers, based on the lever provided at 0.1 ml per drop. The trained rats were divided into EtOH, HT7 + EtOH, and NonAcu + EtOH groups.

### 2.3 Acupuncture stimulation

**Figure 1:**
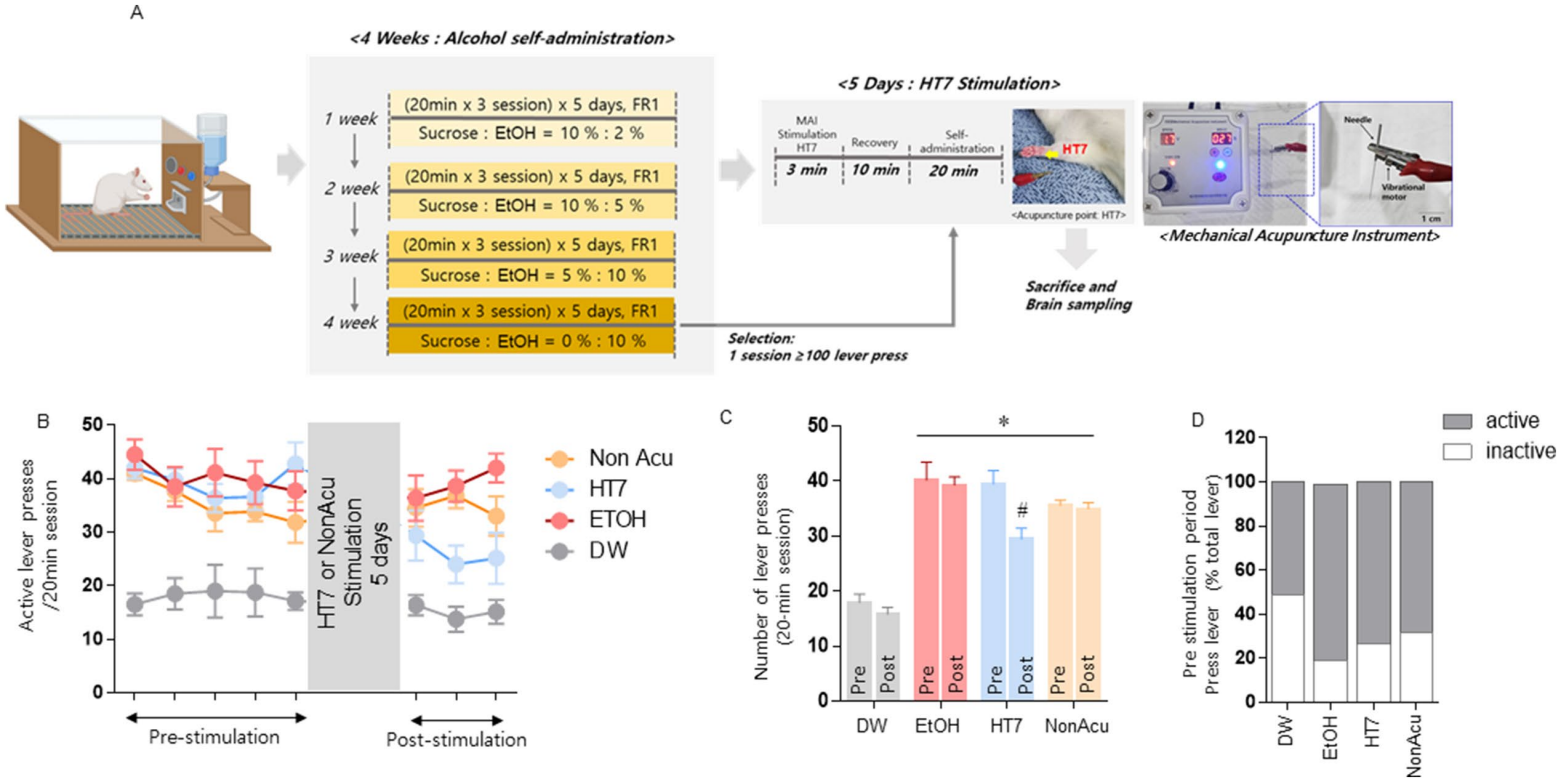
Effect of mechanical acupuncture on alcohol self-administration. The experimental schedule for acupuncture stimulation was performed using MAI at the acupuncture point Shenmen (HT7; **A**). Changes in the number of active lever presses and HT7 stimulation in alcohol self-administering rats (*n* = 8 in each group) were measured 40 min before and 20 min after acupuncture stimulation **(B)**. The changes in the number of active lever presses before and 20 min after acupuncture treatment **(C)**. DW group and EtOH group (EtOH, EtOH + HT7, EtOH + NonAcu) ability to distinguish between active and inactive levers **(D)**. Data were analyzed using repeated measures, two-way ANOVA, and Tukey’s test. *p < 0.05 vs. Pre TEST, ^#^*p* < 0.05 vs. DW group. Values are expressed as mean ± SEM.

Traditional manual acupuncture is the act of inserting a needle into the acupuncture point, sometimes twisting the needle. A mechanical acupuncture instrument (MAI) was developed to provide quantitatively reliable vibration while maintaining the effects of manual acupuncture stimulation (Figure 1A). This equipment was provided by Daegu Oriental Medical University (Daegu, Korea; Kim et al., 2013). The needles (0.18 × 8 mm, Dong Bang Medical, Gyeonggi-do, Korea) were connected to the MAI. For experiments, the MAI was set at an intensity of 1.3 m/s^2^ and a frequency of 85 Hz. The acupuncture point HT7 and a non-acupuncture point (NonAcu) located on the upper part of the left buttock were used in this study. The HT7 acupoint is situated at the ulnar end of the wrist crease when the palm faces upward, on the radial side of the flexor carpi ulnaris tendon. All rats practiced for at least 1 week before starting the study to become accustomed to the movements for acupuncture treatment. Specifically, the acupuncturist grabbed for 1 min and touched the acupuncture points with the acupuncturist’s fingertips. The MAI was applied bilaterally at the acupuncture points and the tail for 30 s and maintained for up to 1 min after needle insertion.

### 2.4 Microinjection treatment

General anesthesia was administered by intraperitoneal injection of Rumpun (0.1 ml, Bayer, Korea) and Zoletil (0.4 ml, Zoletil 50, Virbac Lab, Carros, France). During deep anesthesia, the rats’ heads were placed in a stereotaxic apparatus (Stoelting; Wood Dale, IL, USA). The skull was exposed, and a drill (35,000 rpm; Saeshin Dental Lab, South Korea) was used to drill a hole to implant a cannula into the mPFC (AP = +3.4 mm; ML = −0.8 mm; DV = −5 mm) and Hb (AP = −3.9 mm; ML = ±1 mm; DV = −4.7 mm) according to the stereotactic coordinates of the rat brain atlas (1998 edition; Riga et al., 2016; Casanovas et al., 1999). After surgery, lidocaine (Laboratorios PISA, Mexico City, Mexico) was applied topically to the area surrounding the cannula implant. For the first 3 days after surgery, an analgesic agent (dipyrone 50, Virbac, Guadalajara, Mexico) was administered intraperitoneally to reduce pain. After a week of recovery, the operated mice were reintroduced into the alcohol self-administration chamber. The vehicle was injected through the stainless steel cannula, which was connected to a 5-μl Hamilton syringe by means of a polyethylene tube. An automatic infusion pump (KD Scientific, Holliston, MA, USA) was used to microinject 2 μl of the vehicle over 5 min (speed: 0.4 μl/min). Rats were able to move freely during this procedure. Rats were left to rest for 5 min after microinjection to prevent the vehicle from returning by capillarity. Products used for microinjection include Simga 1 receptor antagonist; BD1047 (#0956, Tocris Bioscience, Bristol, UK), BDNF (#ab9794, Abcam, Cambridge, MA, USA) and microglia inhibitor; This is Minocycline (#3268, Tocris Bioscience). The concentrations for microinjection were carefully determined based on previous studies. The BDNF doses were determined to be 0.5, 5, and 10 nmol based on previous studies (Bekinschtein et al., 2008). The BD1047 concentrations were determined to be 50, 100, and 200 nmol based on previous studies (Roh et al., 2008). The MINO concentrations were also determined to be 50, 100, and 200 nmol based on previous studies (Velasquez et al., 2014; Rojewska et al., 2014).

### 2.5 Western blotting

**Figure 2:**
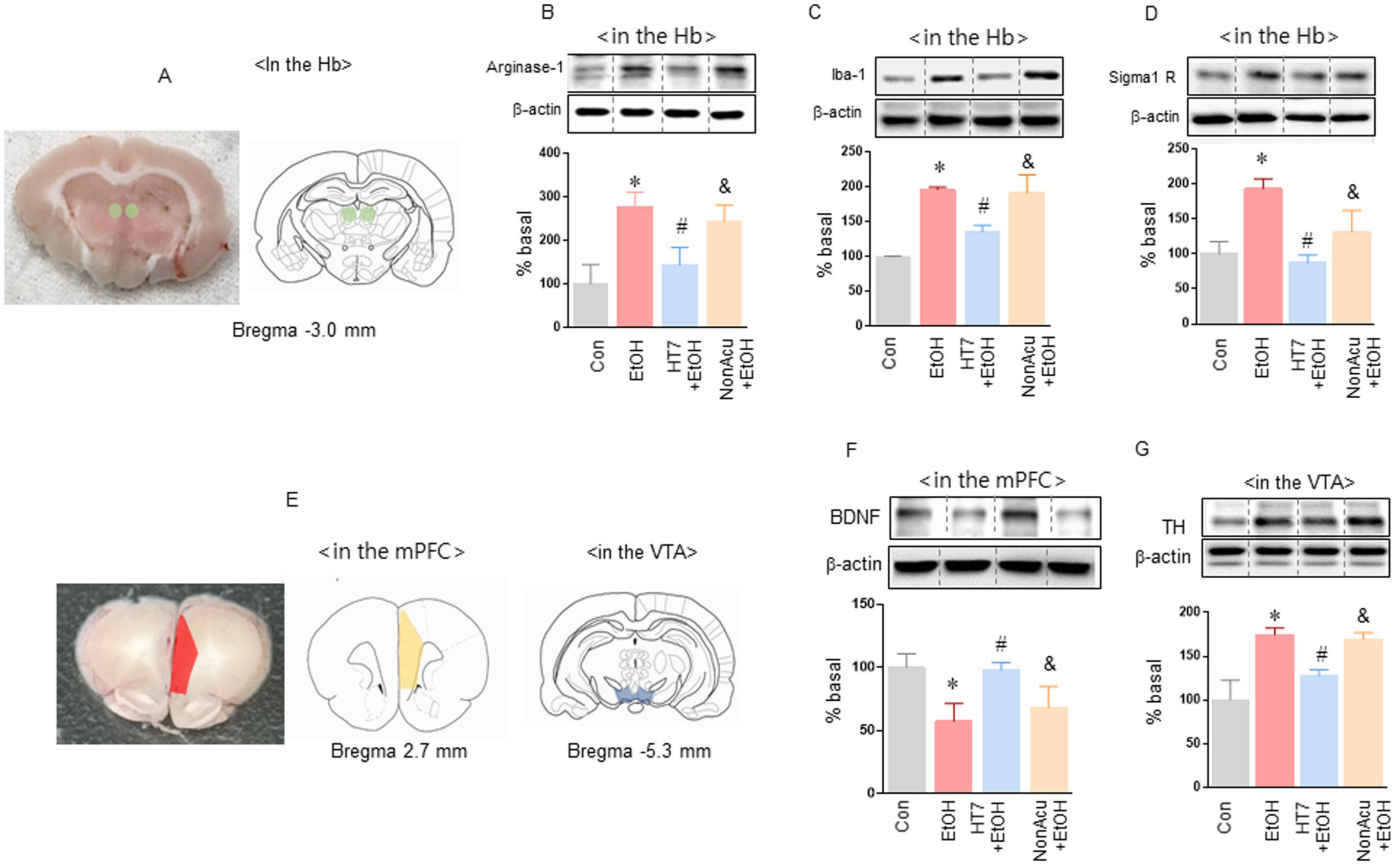
Analysis of protein changes in Hb, mPFC, and VTA caused by acupuncture treatment in HT7. Effect of acupuncture stimulation on microglial activity and sigma 1 receptor levels in Hb **(A)**. Effect of HT7 stimulation on microglial marker (arginase-1, Iba-1 and sigma 1 receptor) expression in Hb of alcohol-preferring rats (*n* = 3–4 for each group; **B–D**). Effect of acupuncture stimulation on BDNF and TH level in mPFC and VTA. The effects of mechanical acupuncture on BDNF expression in the mPFC in rats exposed to alcohol self-administration (*n* = 3–4 for each group; **E,F**). The effects of mechanical acupuncture on TH expression in the VTA (*n* = 3–4 for each group; **E,G**). Data were analyzed using repeated measures ANOVA and Tukey test. *p < 0.05 vs. Con group and ^#^*p* < 0.05 vs. EtOH group and ^&^*p* < 0.05 vs. HT7 + EtOH group. Values are expressed as mean ± SEM.

**Figure 3:**
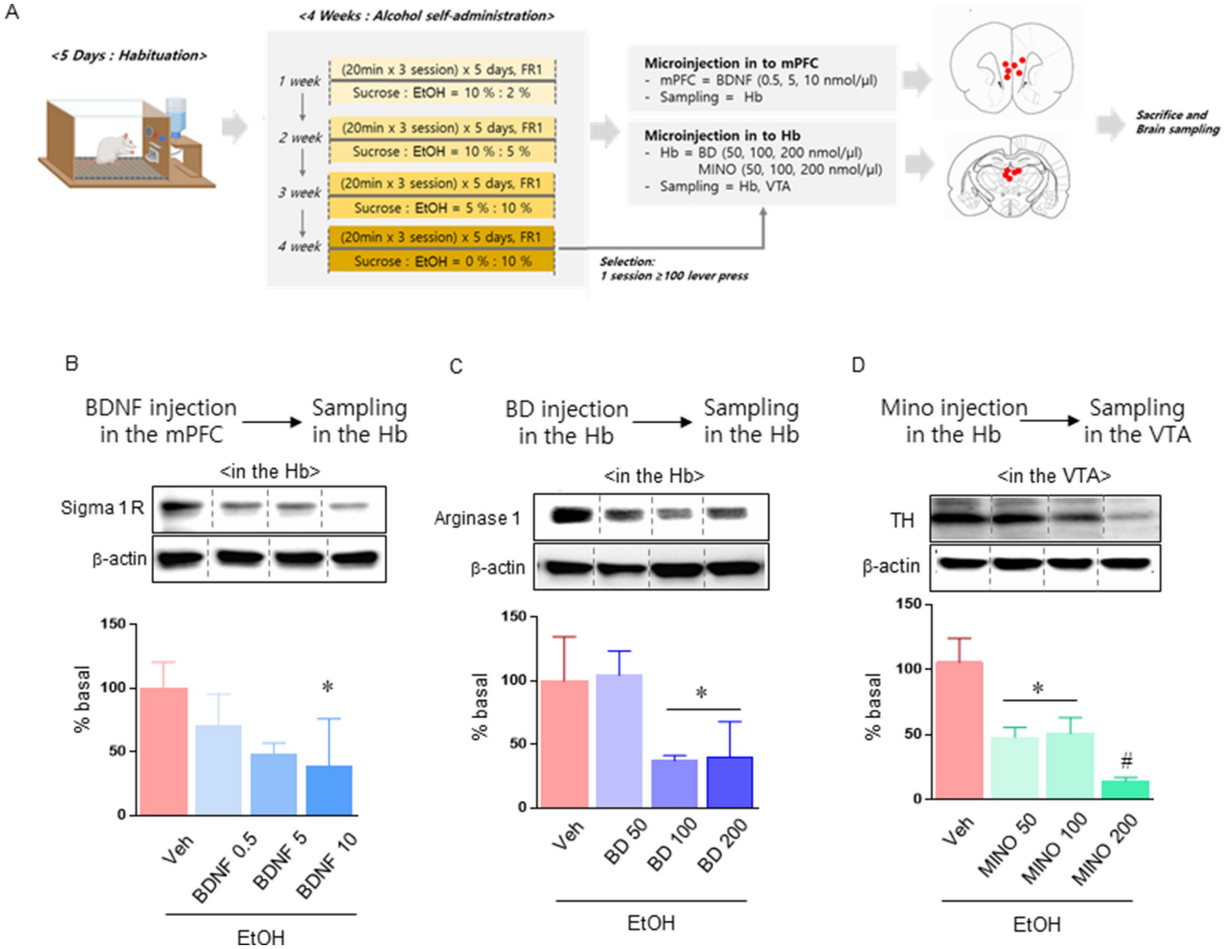
Experiment to confirm the effect mechanism of acupuncture treatment. Schematic showing the experimental schedule of the effects of BDNF and BD microinjections in mPFC and Hb **(A)**. Sigma 1 receptor expression levels in the Hb after microinjection of BDNF into the mPFC (*n* = 3–4 in each group; **B**). Arginase 1 levels in the Hb after microinjection of BD into Hb (*n* = 3–4 in each group; **C**). TH levels in the VTA after microinjection of MINO into Hb (*n* = 3–4 in each group; **D**). Data were analyzed using repeated measures ANOVA and Tukey test. *p < 0.05 vs. Veh group and ^#^*p* < 0.05 vs. MINO100 group. Values are expressed as mean ± SEM.

**Figure 4:**
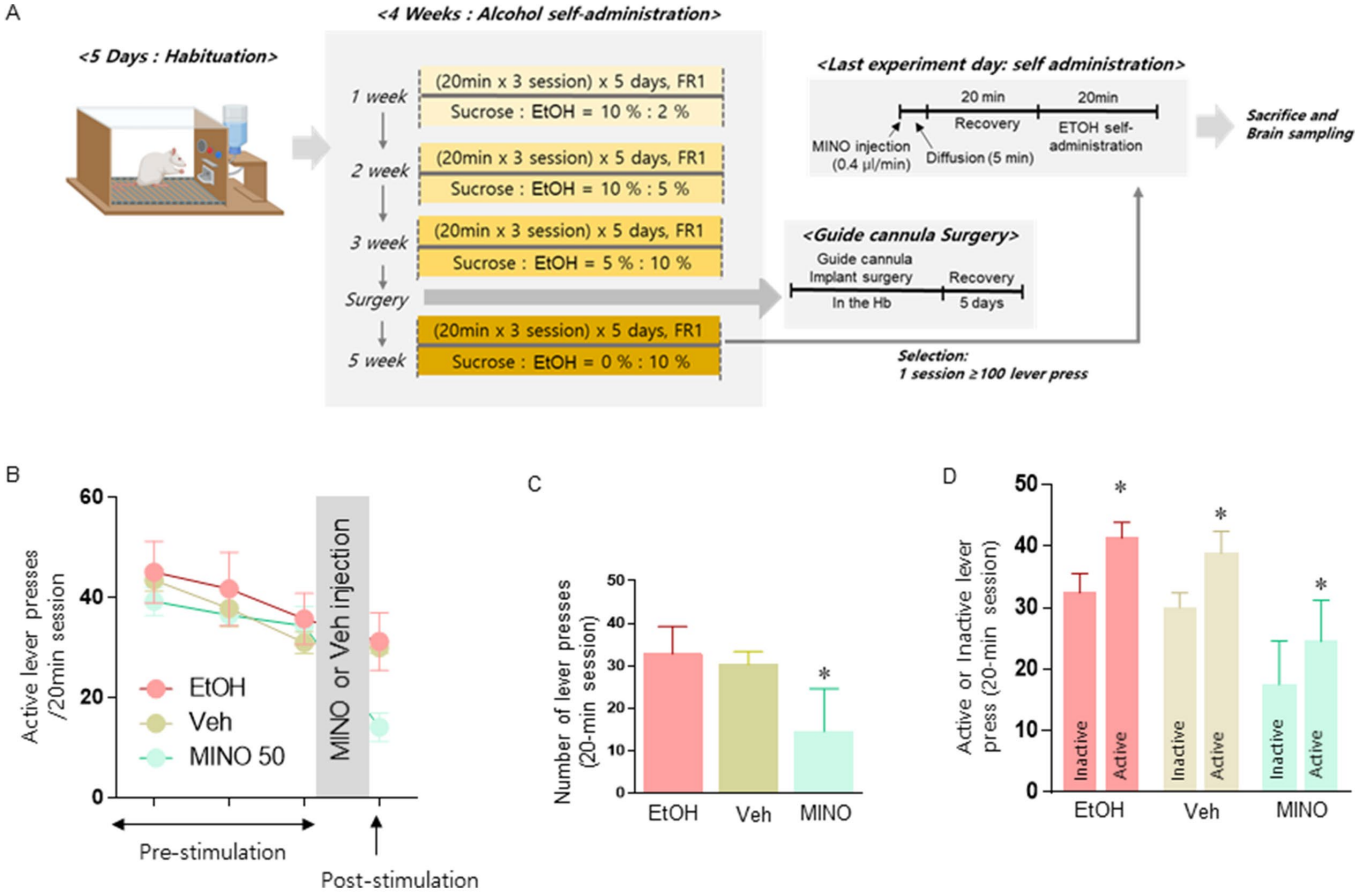
Schematic diagram showing the experimental schedule to determine the effect of Hb on the actual number of alcohol levers when suppressing microglia **(A)**. Microinjection of the microglial inhibitor MINO into Hb restores TH levels in the VTA (*n* = 8 for each group; **B**). Changes in the number of active lever presses after microinjection of MINO into the Hb of rats exposed to alcohol self-administration (*n* = 8 in each group; **C**). Comparison of the number of active lever and inactive lever presses of each group of rats (*n* = 5 in each group; **D**). **(C,D)** Data were analyzed using repeated measures two-way ANOVA and Tukey test. *p < 0.05 vs. Veh group. **(D)** Data were analyzed using repeated measures two-way ANOVA and Tukey test. *p < 0.05 vs. inactive lever group. Values are expressed as mean ± SEM.

**Figure 5:**
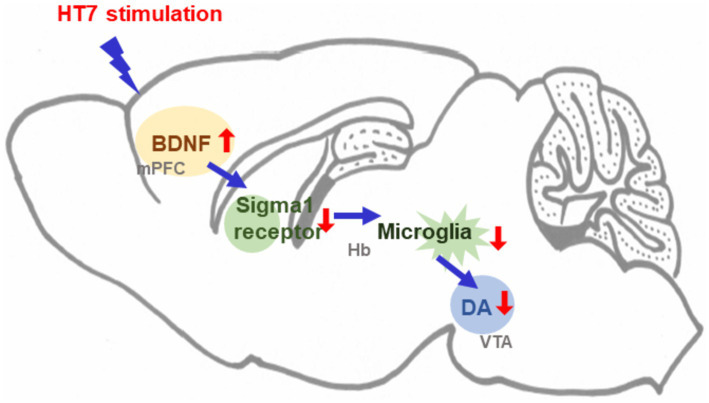
Schematic diagram illustrating the mechanism underlying the effects of acupuncture stimulation.

Twenty minutes after the final experiment, the rats were anesthetized with N2O/O2 gas and then sacrificed. The brains extracted from the rats were continuously cut into sections using a rat brain matrix, and the brain regions that were needed for the subsequent analyses were then selected. The brain samples were lysed in RIPA buffer, sonicated for 50 s, and incubated for 1 h on ice. The brain samples were precipitated by centrifugation at 13,000 rpm for 60 min, and the protein contents in the supernatants were assayed using the Bradford method. The samples were electrophoretically separated on 4–10% bis-Tris gels and then transferred to a nitrocellulose membrane. All membranes were cut and used prior to hybridization with the antibody during blotting. The membrane was blocked with a blocking reagent (TOYOBO Life Science, Osaka, Japan). Membranes were incubated with primary antibodies (anti-BDNF; Abcam, ab108319, anti-Argimase-1; Invitrogen, Carlsbad, CA, USA, 702730, anti-Iba-1; abcam, ab108539, anti-sigma 1 receptor; abcam, ab307548, anti-TH; abcam, ab315252, and anti-β-actin; Santa Cruz Biotechnology, CA, sc-517582) at a dilution of 1:1000 in blocking solution in PBS overnight at 4°C. The next day, the membrane was washed and incubated with secondary antibodies for 1 h at room temperature. The secondary antibody was horseradish peroxidase (HRP)-labeled goat anti-rabbit or HRP-labeled goat anti-mouse (1:1000, KPL, Gaithersburg, MD, USA). The protein bands on the membrane were exposed to enhanced chemiluminescence reagents (Thermo Fisher). The fluorescence-amplified signals on the membrane were extracted as an image using a Fusion SL4 imaging system (Vilber Lourmat, Eberhardzell, Germany), and the results were quantified using ImageJ software.^1^ After the Western blot on the samples, a representative band was selected and shown in Figures 2–5.

### 2.6 Statistics

All data are expressed as the mean ± the standard error of the mean (SEM). Statistical analyses were performed using Prism 6 (GraphPad Software, San Diego, CA, USA). For the alcohol intake analysis, data were compared using a two-way or one-way analysis of variance (ANOVA). *Post hoc* analysis was performed using Tukey’s multiple comparison test and Bonferroni test to determine differences in values among experimental groups. Statistical significance was considered when *p* < 0.05.

## 3 Results

### 3.1 Effect of mechanical acupuncture treatment on alcohol self-administration rats

In this experiment, we investigated whether stimulation of the HT7 acupoint changes the number of alcohol acquisition levers. We trained animals to self-administer alcohol orally using a modified sucrose fading method established in past studies. Rats that completed 10% alcohol training were simultaneously subjected to vibratory needle treatment for 5 days. On the last day of acupuncture treatment, the rats recovered after acupuncture treatment (total acupuncture treatment and recovery period of 20 min) and were placed in an alcohol self-administration room. The number of lever presses was checked (Figure 1A). As a result, there was a statistically significant decrease in the number of times the alcohol self-administration lever was pressed for more than 20 min. However, there was no significant difference in the NonAcu group (Figure 1B). As a result of examining the average number of lever presses before and after acupuncture treatment, it was confirmed that the number of lever presses decreased due to acupuncture treatment. Additionally, as a result of checking the number of active and inactive lever presses, the number of active levers was lower in the EtOH, HT7, and NonAcu groups compared to the DW (Distilled Water) group that did not receive any treatment [Group: *F* (1, 54) = 6.246, *p* = 0.0155, treatment: *F* (3, 54) = 55.85, *p* < 0.0001; Figure 1C]. It was confirmed that the ability to distinguish between active and inactive levers before acupuncture stimulation was well learned (Figure 1D). This means that learning about alcohol self-administration was successful. These results demonstrate that acupuncture stimulation treatment can reduce the frequency of alcohol self-administration.

### 3.2 Stimulation at HT7 reduced the increased microglial activity and sigma 1 receptor level of the Hb in the alcohol self-administration rats

Next, we measured microglial activity and sigma 1 receptor activity in rat Hb (Figure 2A). Alcohol self-administration significantly increased Arginase-1 levels in Hb. Stimulation of HT 7 by mechanical acupuncture but not NonAcu stimulation group significantly reduced Arginase-1 levels in Hb in the alcohol self-administration group, [treatment: *F* (3, 10) = 16.26, *p* = 0.0004; Figure 2B]. Iba-1 levels also increased in the alcohol self-administration group. And HT7 stimulation significantly reduced Iba-1 levels in Hb in the alcohol self-administration group [treatment: *F* (3, 22) = 12.88, *p* < 0.0001; Figure 2C]. sigma 1 receptor levels of Hb were increased in the alcohol self-administration group. However, HT7 stimulation significantly reduced sigma 1 receptor levels in Hb in the alcohol self-administration group [treatment: *F* (3, 14) = 20.98, *p* < 0.0001; Figure 2D]. Stimulation at HT7 increased the BDNF level in the mPFC and decreased TH in the VTA in alcohol self-administration rats.

### 3.3 Stimulation of HT7 restored the expression of BDNF and TH in the mPFC and Hb of alcohol self-administration rats

In the next experiment, we measured the expression levels of BDNF in the mPFC and TH in the VTA when alcohol self-administering rats were stimulated (Figure 2E). Alcohol self-administration significantly reduced BDNF levels in the mPFC. Stimulation of HT7 by mechanical acupuncture significantly increased BDNF levels in the mPFC in the alcohol self-administration group HT7 [treatment: *F* (3, 12) = 11.42, *p* = 0.0008; Figure 2F]. In addition, the expression of TH in the VTA was measured when HT7 was stimulated in alcohol self-administration rats. Alcohol self-administration significantly increased TH expression in the VTA. Stimulation of HT7 by mechanical acupuncture significantly reduced TH expression in the VTA in the alcohol self-administration group HT7 [treatment: *F* (3, 10) = 29.11, *p* < 0.0001; Figure 2G].

### 3.4 Verification of protein activity level in each region of mPFC-Hb-VTA to determine the effect of HT7 stimulation on restoration alcohol self-administration effects in the rats

In the previous experiment, we confirmed that alcohol self-administration resulted in a decrease in BDNF in the mPFC, an increase in Hb glial activity and sigma 1 receptor activity, and an increase in TH expression in the VTA. In the next experiment, we performed microinjection into the corresponding region of the circuit and performed protein quantitative analysis to confirm the cascade effect of mPFC, Hb, and VTA (Figure 3A). We administered BDNF to the mPFC to determine how increasing BDNF levels in the mPFC affected sigma 1 receptors in Hb. Administration of high doses (10 nmol/μl) of BDNF significantly reduced sigma 1 receptor levels in HT7 [treatment: *F* (3, 10) = 3.793, *p* = 0.0473; Figure 3B]. In the next experiment, we determined how Hb’s sigma 1 receptor activity affected Arginase-1 levels. Administration of high doses (100, 200 nmol/μl) of BD significantly reduced sigma 1 receptor levels in HT7 [treatment: *F* (3, 10) = 7.876, *p* = 0.0055; Figure 3C]. Finally, MINO was administered to determine whether arginase expression in Hb affects TH expression in the VTA. Administration of MINO at all concentrations we selected affected TH expression in the VTA [treatment: *F* (3, 10) = 32.05, *p* < 0.0001; Figure 3D].

### 3.5 Inhibiting microglia in the Hb reduced the expression of TH in the VTA and decreased alcohol self-administration

We tested whether microinjection of 50 nmol/μl MINO had an inhibitory effect on alcohol intake in rats in a self-administration operant chamber (Figure 4A). Compared to the microinjection of the vehicle, microinjection of 50 nmol/μl MINO into Hb significantly reduced the number of active lever presses for alcohol delivery [treatment: *F* (2, 14) = 11.04, *p* = 0.0013; Figures 4B,C]. Considering that the active lever and inactive lever are clearly distinguished, it can be seen that the experiment was conducted with a well-established alcohol self-administration model [Group: *F* (1, 23) = 15.46, *p* = 0.0007, treatment: *F* (2, 23) = 21.32, *p* < 0.0001; Figure 4D].

## 4 Discussion

In this study, we found that the number of active lever presses in alcohol self-administering rats was reduced by mechanical acupuncture with HT7 (also known as “Shenmen”). Alcohol self-administration increased the activity of both types of microglia and increased the expression of sigma 1 receptors in the Hb. Repetitive HT7 stimulation reduced microglial activity, increased alcohol intake, and attenuated the expression of sigma 1 receptors. Meanwhile, alcohol self-administration decreased the expression of BDNF in the mPFC and increased the expression of TH in the VTA. HT7 stimulation increased BDNF expression and decreased TH levels. Based on these results, we investigated the signaling mechanism of activation of protein in the mPFC, Hb, and VTA regions regulated by HT7. BDNF microinjection in the mPFC inhibited the activity of sigma 1 receptors in Hb. Microinjection of BD, an antagonist of the sigma 1 receptor, into Hb, decreased the expression of arginase 1. Additionally, the administration of microglial inhibitors to Hb inhibited the expression of TH in the VTA. Finally, it was confirmed that alcohol self-administration response decreased after MINO was administered to Hb. As a result, HT7 stimulation increases BDNF expression in the mPFC and modulates microglial activity in the Hb region. The decrease in Hb microglia activity restores the level of the sigma 1 receptor, which is increased by alcohol and ultimately regulates the level of TH expression in the VTA. Through this response, HT7 stimulation induces a reaction to alcohol that reduces the number of active levers. This study demonstrates that acupuncture at the HT7 acupoint is an effective non-pharmacological approach to reducing alcohol self-administration, contributing to the limited research on such interventions.

Rodent models of alcohol use disorder closely reflect human behavior and neural mechanisms, particularly in compulsive drinking and withdrawal symptoms. To investigate the effects of acupuncture on alcohol use disorder, we conducted alcohol self-administration tests in rats. Our results showed that 10% (v/v) alcohol self-administration significantly increased the number of active lever presses in alcohol self-administration rats compared to the administration of water. Stimulation at HT7 for 5 days significantly reduced the number of active lever presses increased by alcohol self-administration. This result is consistent with previous reports that the administration of mechanical acupuncture at HT7 significantly decreased 10% (v/v) alcohol consumption compared with the vehicle treatment (Kang et al., 2017). Based on these results, we investigated which brain circuits HT7 stimulation modulates to reduce the number of lever presses active in alcohol self-administration.

One of the brain regions that has recently received attention in addiction research is the Hb. The lateral Hb plays an important role in alcohol use disorder and influences both the rewarding and aversive properties of alcohol (Velasquez et al., 2014). Manipulating LHb function can ameliorate recurrent drinking and psychiatric disorders in abstinent animals, suggesting that alcohol use disorders are related. It is known that short-term and long-term exposure to alcohol increases the expression of inflammatory cytokines in various parts of the brain and causes neuronal death (Adams et al., 2020). For this reason, we confirmed the expression of microglial cells in the habenula area and the expression of the sigma 1 receptor, which controls the activity of microglial cells. Our results show that alcohol self-administration significantly increased the expression of microglial markers (Arginase-1 and Iba-1) and sigma 1 receptor in Hb. Stimulation of HT7 by mechanical acupuncture decreased Arginase-1 Iba-1 and sigma 1 receptor levels in Hb. In other words, it can be seen that the decrease in the number of alcohol activity levers due to HT7 stimulation occurs through the regulation of Hb cytokines and sigma 1 receptor activity.

In the next experiment, we investigated the upper- and lower-level mechanisms that regulate Hb activity. A candidate brain region thought to influence microglial activity in the habenula is the mPFC (Kim and Lee, 2012). The mPFC is a representative region that sends glutamatergic neuron projections to the habenula (Koob and Volkow, 2010; Stephens and Duka, 2008). Based on the findings that the level of BDNF in the mPFC increases by acupoint stimulation, we investigated the level of BDNF in the mPFC as an upstream mechanism regulating Hb. Meanwhile, the mesolimbic dopamine system is thought to play an essential role in mediating the positive reinforcing effects of alcohol and other drugs of abuse (Gonzales et al., 2004; Stuber et al., 2012). One of the mesolimbic dopamine systems, the VTA, has a well-studied association with Hb. Abnormalities in LHb result in excessive inhibition of downstream dopamine neurons, which has the potential to cause various emotional disorders (Bao and Zheng, 2023). Alcohol is known to activate LHb neurons that project to the VTA, RMTg, and raphe (Fu et al., 2017). For this reason, we investigated DA neurons in the VTA as a downstream stream affected by microglial activation of Hb. Our results showed that BDNF in the mPFC was reduced by alcohol self-administration. The decreased BDNF expression level was restored by HT7 stimulation. Meanwhile, TH expression in the VTA was confirmed to increase upon self-administration of alcohol. HT7 stimulation reduced the increased TH activity. In other words, our results allow us to infer the mPFC-Hb-VTA activation circuit caused by alcohol self-administration.

To determine whether the mPFC-Hb-VTA circuit we inferred was activated by alcohol self-administration, we probed the circuit using microinjections. To confirm whether the increase in BDNF in the mPFC region inhibits the activity of the sigma 1 receptor in the Hb region, BDNF was injected at different concentrations into the mPFC. Then, the expression of the sigma 1 receptor in the Hb region was examined. As a result, it was confirmed that BDNF expression in mPFC inhibits the activity of the sigma 1 receptor in Hb. In the next experiment, BD 1047, an antagonist of the sigma 1 receptor, was injected into Hb to determine whether the sigma 1 receptor in the Hb activity regulated microglial activity. As a result, it was confirmed that inhibiting the activity of the sigma 1 receptor can inhibit the activity of microglial cells. Finally, MINO was injected into Hb to determine whether inhibition of microglial activity in the Hb region regulates TH expression in the VTA. As a result, it was confirmed that TH expression in the VTA was decreased. Our results are consistent with various previous studies showing that microglia are regulated by sigma 1 receptor activity. For example, It is known that the sigma 1 receptor regulates cytosolic calcium in microglia (Schober et al., 2023). Additionally, the sigma 1 receptor is known to reduce microglial activity through the regulation of ER stress and cell death mechanisms(Chao et al., 2017; Ruiz-Cantero et al., 2021; Shi et al., 2022). Additionally, consistent with the finding that MINO (50 mg/kg), a microglial inhibitor, significantly reduced alcohol intake in male and female C57Bl/6 J mice using a free-choice voluntary drinking model(Agrawal et al., 2011) as well as MINO (50 mg/kg/day, 4 days) is also consistent with a previous study that reduced alcohol intake in a binge drinking model (Lainiola and Linden, 2017). In other words, it can be confirmed that the activity of each region corresponding to the mPFC-Hb-VTA circuit is related to each other. Finally, to determine whether this circuit actually affects the number of alcohol-activated levers, we examined whether the administration of MINO, the final step in the circuit, induces changes in the number of levers. Because the regulation of microglia activation by Sigma 1 receptor was clear in previous studies, combined treatment with BD and MINO was not considered in the alcohol lever-pressing experiment. As a result, the number of lever presses increased by alcohol, but the number of lever press responses decreased after MINO administration. In other words, it can be seen that the mPFC-Hb-VTA circuit contributes to controlling the number of alcohol levers. Although we could not verify it experimentally in our study, the reason why mPFC-Hb-VTA circuit activity contributes to the control of alcohol lever number is the recovery of the mesolimbic dopaminergic system by activating DA neurons in the VTA through acupuncture treatment, and the DA neurons in the VTA. We predict that regulation of mu-opioid activity in the VTA by activation may be involved.

The key mechanism of acupoint stimulation is to regulate overall homeostasis within the brain. Various methods to modulate DA neuron activity in the VTA have been studied to control alcohol use disorder. Acupuncture is also known to regulate the activity of DA neurons in the VTA (Lee et al., 2021). A representative example is the PFC-NAc-VTA circuit (Vornholt et al., 2020). This study additionally identified a pathway that regulates the activity of DA neurons in the VTA in addition to what was previously known. Meanwhile, the primary limitation of our study is the focus on the mPFC-Hb-VTA circuit, with the identification and validation of only a select number of proteins. However, alcoholism induces complex and widespread effects across multiple brain regions. Thus, additional studies are required to comprehensively investigate BDNF and microglial activity in various brain regions following acupuncture treatment and to elucidate how alterations in their expression influence TH neuron activity in the VTA.

## 5 Conclusion

This study demonstrates that mechanical acupuncture at the HT7 acupoint effectively reduces alcohol self-administration in rats by modulating the mPFC-Hb-VTA circuit. HT7 stimulation decreases microglial activity and sigma 1 receptor expression in the Hb, increases BDNF levels in the mPFC, and TH expression in the VTA. These findings suggest that HT7 acupuncture exerts its therapeutic effects on alcohol use disorder by regulating neuroinflammatory pathways and neurotransmitter systems, thereby providing a promising non-pharmacological treatment strategy for alcohol use disorder (Figure 5). These molecular changes may contribute to the long-term prevention of alcohol use disorder relapse by readjusting the neural circuits associated with alcohol dependence. By suppressing alcohol-induced neuroinflammation and restoring balance in the dopamine system, HT7 acupuncture has the potential to serve as an effective non-pharmacological treatment in the long term. Compared to existing non-pharmacological treatments, this study uniquely positions HT7 acupuncture as a targeted intervention, broadening the therapeutic options for alcohol use disorder.

## Data Availability

The datasets presented in this study can be found in online repositories. The names of the repository/repositories and accession number(s) can be found in the article/Supplementary material.

## Footnotes

^1^ http://rsb.info.nih.gov/ij/
