# Supplementary figures and images for: Mechanical acupuncture at HT7 attenuates alcohol self-administration in rats by modulating neuroinflammation and altering mPFC-habenula-VTA circuit activity
Source: Front Behav Neurosci. 2024 Oct 30;18:1455622. doi: 10.3389/fnbeh.2024.1455622 (PMC11557434; doi:10.3389/fnbeh.2024.1455622)

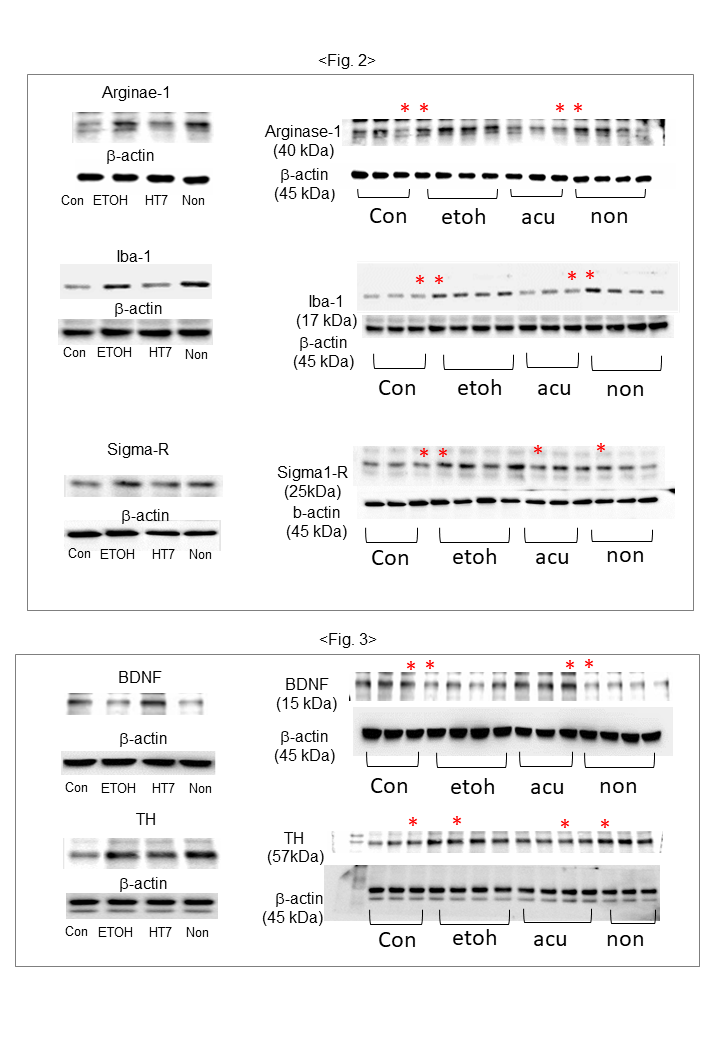

Supplement: Supplementary file 1 [file Image_1.TIF]

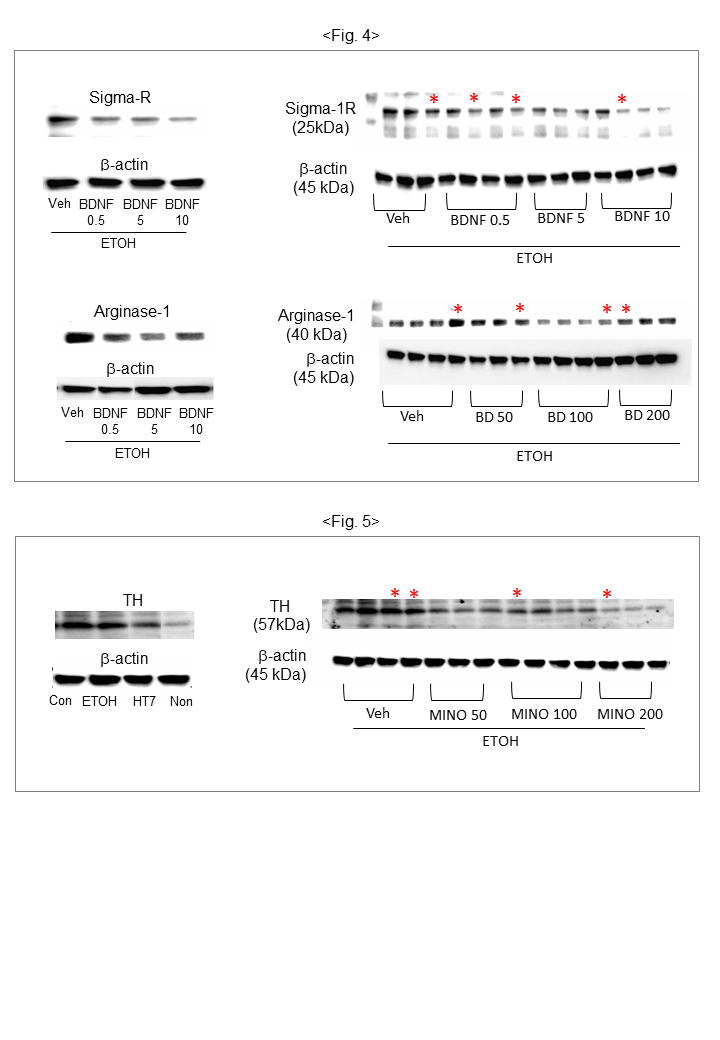

Supplement: Supplementary file 2 [file Image_2.TIF]
